# Steroidal Alkaloids as an Emerging Therapeutic Alternative for Investigation of Their Immunosuppressive and Hepatoprotective Potential

**DOI:** 10.3389/fphar.2017.00114

**Published:** 2017-03-21

**Authors:** Naeem U. Jan, Bashir Ahmad, Safdar Ali, Achyut Adhikari, Amjad Ali, Azra Jahan, Abid Ali, Hamid Ali

**Affiliations:** ^1^Center of Biotechnology and Microbiology, University of Peshawar, PeshawarPakistan; ^2^Pakistan Institute of Engineering and Applied Sciences, IslamabadPakistan; ^3^Hussain Ebrahim Jamal Research Institute of Chemistry, International Center for Chemical Sciences, University of Karachi, KarachiPakistan; ^4^Faculty of Biological Sciences, Department of Biochemistry, Quaid-i-Azam University, IslamabadPakistan; ^5^Department of Zoology, Abdul Wali Khan University, MardanPakistan; ^6^Laboratory of Germplasm Innovation and Molecular Breeding, Department of Vegetable Sciences, College of Agriculture and Biotechnology, Zhejiang University, HangzhouChina; ^7^Department of Biosciences, COMSATS Institute of Information Technology, IslamabadPakistan; ^8^Dr. Panjwani Center for Molecular Medicine and Drug Research, International Center for Chemical and Biological Sciences, University of Karachi, KarachiPakistan

**Keywords:** steroidal alkaloids, hepatoprotection, immunosuppressive, IL-2, T-cells, cytotoxicity

## Abstract

The compounds, sarcovagine-D, alkaloid-C, and holaphylline isolated from *Sarcococca saligna* were found to possess immunosuppressive activities. These compounds were characterized for *in vitro* inhibition on human T-cells proliferation and IL-2 production. The compounds showed significant immunosuppressive effect on IL-2 production as well as on phytohemagglutinin stimulated T-cell proliferation in a dose dependent manner. Of all the tested compounds holaphylline was found to be less toxic and safe. These compounds were then evaluated for their *in vivo* hepatoprotective potential against CCl_4_, in which alkaloid-C and holaphylline showed markedly reduced liver inflammation and biochemical parameter (ALT, AST, and ALP) of liver injury. The decrease in the activity of hepatic antioxidant enzyme (SOD) was significantly prevented by holaphylline, likewise gradually the levels of MDA and GSH were also normalized compared to silymarin. The CCl_4_ induced inflammation and necrosis around the central vein of liver was reduced by sarcovagine-D, alkaloid-C and holaphylline, to 8%, 4% to 1% respectively as assessed by histopathology, thus having better hepatoprotective effect compared to positive control. Steroidal alkaloids attenuated the inflammation of liver around the injured central vein region by down regulating the CCl_4_ induced activation of hepatic macrophages as well as their number respectively. Therefore, the *in vitro* and *in vivo* results suggest that steroidal alkaloids from *S. saligna* could be excellent immunosuppressive and hepatoprotective agents.

## Introduction

The liver plays an important role in the innate immune response thereby providing the first line of defense against microbes and toxins. The liver is one of the most vital organs in human body responsible for metabolism and therefore it is more vulnerable to injury which can produce different diseases like hepatitis, cirrhosis, or hepatocellular carcinoma. Different environmental pollutants and drugs or chemicals are the major cause of these diseases. There are about 550 million people worldwide infected from hepatitis (Alter, 2006) and about 35 million people are infected in Pakistan (André, 2000) from this disease. The liver injury induced by many drugs causes inflammation and mortality around the world (Ghabril et al., 2010).

Macrophages upon injury produces a number of inflammatory cytokines such as TNF-α that leads to various conditions such as inflammation, allergy, or autoimmune diseases in different organs (Thomas and Donald, 2005). Acute liver inflammation occurs through infiltration of inflammatory cells such as macrophages, T-cells and neutrophils (Heijerman, 2005). The CD4^+^T-cells produced interleukin-2 in response of xenobiotic through onset of TCR and MCH I and II molecules (major histocompatibility complex) of the surface of antigen presenting stimulated cells (Malek et al., 2008). The IL-2 level is almost undetectable in normal healthy human but it rises quickly when a person exposed to infection or injury.

The liver has kupffer cells which are actively involved in the elimination of microorganisms from the blood (Gregory and Wing, 1998; Nagy, 2003). Kupffer cells are also present at the damaged area in the liver along with other inflammatory cells. Microorganisms, drugs and other chemicals cause activation of monocytes, neutrophils, lymphocytes, and natural killer cells. Kupffer cells during infection produces neutrophilic mediators including oxidative species, necrosis factor (TNF-α), interleukins and chemokines (Sweet and Hume, 1996). The chronic injury of liver will produced active neutrophils through inflamed mediators in the microvasculature of liver, which produces different oxidative stress factors causing hepatocellular death.

To treat such inflammatory condition there is need of an effective development of anti- inflammatory agent which have less side effect or harmless to immune system. The use of synthetic drugs for such diseases have severe adverse effect and therefore natural drug or bioactive compounds from herbal source can be used for such disease which have less side effects. Studies suggest that steroidal alkaloids isolated from plant have anti-inflammatory and hepatoprotective effect acting as an antioxidant and free radical scavenging properties (Ali et al., 2015).

To investigate chemical induced oxidative stress-mediated hepatotoxicity, carbon tetrachloride (CCl_4_) is used widely in animal model (Recknagel and Glende, 1973). Chronic liver injury induced by CCl_4_ has similar symptoms as in human chronic liver injury (Basu, 2003). The liver injury occurs through cytochrome-p450, produce different types reactive oxygen species (ROS) by CCl_4_ (Tada et al., 2003). Lipid peroxide produced from different radical were formed by binding of ROS with polyunsaturated fatty acid, causes membrane damage and changes in enzyme activity (Weber et al., 2003), which would increase hepatic injury, inflammation, necrosis and apoptosis of hepatocytes (Lin et al., 2009).

*Sarcococca saligna* (D. Don) Muell belong to Buxaceae family is an evergreen dicotyledonous shrub with a scaly buds, found in areas of high altitudes mountains of Pakistan like Swat, Dir, Manshera, Kashmir, and other northern regions. Traditionally the leaves and shoots of this plant were used for stomach disorder, blood disorder and also for muscles aching (Ahmad et al., 2015). The steroidal alkaloids extracted from these species are pharmacologically active and has shown different biological activities. Steroidal alkaloid, salignine from *S. saligna* have shown ganglion blocking activity on guinea pig ileum and partially blocking in cat. It also increases the contractile action of acetylcholine in rat diaphragm through blocking reversibly cholinesterase enzyme activity. The LD-50 value of salignine showed less toxicity than pysostigmine and neostigmine (Harris et al., 2009). *S. saligna* extract has been widely used against pain, malaria, rheumatism and skin infections diseases (Mollazadeh et al., 2010). The alkaloid isolated from methanolic extract of *S. saligna* showed cardio-suppressant, vasodilator, and tracheal relaxant activities (Ghayur and Gilani, 2006). Steroidal alkaloidal compounds isolated from *S. saligna* have been found to possess antibacterial, antileishmanial and a potent acetylcholinestrase enzyme inhibitory activity which can be used in several nervous disorders (Ojha et al., 2003). Several steroidal alkaloids isolated from this plant have antitumor and antiulcer activities (Zaheer-Ul-Haq et al., 2003). Some steroidal alkaloids isolated from *S. saligna* have potential of hepatoprotective properties (Ali et al., 2015) while some alkaloids from this plant have potential to stop diarrhea and excessive secretion in mice (Giliani et al., 2005). The aim of present study is to investigate the immunosuppressive and hepatoprotective activity of steroidal alkaloids, sarcovagine-D, alkaloid-C, and holaphylline from *S. saligna* plant.

## Materials and Methods

### Plant Material

*Sarcococca saligna* (D.Don) Muel whole plant (40 Kg) was collected in June 2014 from Miandam, District Swat, Khyber Pakhtoonkhwa, Pakistan. The plant was identified by Dr. Jilani, a botanist at Department of Botany, University of Peshawar, Pakistan and specimen voucher But.20098(pup) was submitted in the herbarium section of Botany Department.

### Extraction and Isolation

The whole plant (40 Kg) of *S. saligna* was shade dried and crushed in to powder. The powder was soaked in to MeOH/H_2_O mixture ratio 8:2 of 35 lt for 20 days. The methanolic extract was filtered under vacuum and become concentrated (2 Kg). The distilled water (2 lt) was used to solubilize the concentrated methanolic extract. The mixture then defatted with *n*-Haxane (254 gm). When fatty material removed, the aqueous layer then extracted with Chloroform at pH 6 to give extract of chloroform (200 gm). The rest of aqueous fractions extracted with ethyl acetate (150 gm) and butanol finally (100 gm). The Chloroform extract then subjected for further elution through over neutral Al_2_O_3_ column chromatography. The elution took place by increasing polarities of *n*-Hexane/ethyl acetate/diethylamine to get three different fractions (F1–F3) which contain steroidal alkaloids through detection on TLC precoated silica gel and dragendroff spray which shows orange spots. The fractions F2 (3.8 g) were further subjected individually on neutral Al_2_O_3_ column chromatography. The elution took place by increasing polarities of solvents *n*-hexane/ethyl acetate/ with a few drops of diethylamine to get partially pure fractions NA (230 mg). The partially pure fractions NA subsequently subjected for Al_2_O_3_ column chromatography in order to obtained purified steroidal alkaloids compounds. The elution took place by using solvents system of *n*-hexane/ethyl acetate/and few drops of diethylamine for increasing polarities which get the Purified isolated compounds NA-8 (180 mg). Similarly, the purified compound NF23-4 (155 mg) and NF73-31(135 mg) isolated through elution over neutral Al_2_O_3_ column chromatography by increasing solvent system polarities of *n*-hexane/ethyl acetate with a few drops of diethylamine.

Sarcovagine-D (NA-8): Sarcovagine-Doccurs as a white crystalline solid. The spectral data showed that the compound NA-8 (sarcovagine-D) is known and isolated previous from the *S. vagans* (Yu et al., 1997). Alkaloid C, occurs as white amorphous powder. The compound is known by comparing spectral data with the previous literature and isolated first time from the same plant (Giliani et al., 2005). Holaphylline, occur as a sticky light yellowish powder. The spectral data showed that the compound NF-73-31 (holaphylline) is known but first time isolated from this species and previously reported from *Holarrhena floribunda* plant (Quevauviller and Blanpin, 1960).

### Materials

The analytical grade reagents were used for chromatography and detection techniques. Different column sizes were used for chromatography with alumina (Al_2_O_3_) and silica gel (SiO_2_) for separation. TLC plates (Merck GF-254) with precoated SiO_2_ and Dragendroff, s reagent spray for visualization. Hitachi UV-3200 spectrophotometer, IR Jasco A-302 model spectrophotometer, Mass spectrometer, model Jeol HX-110, BrukerAvanceAM-400 and AC-300 NMR spectrometer were used for the current study.

The material and instrument used for assay were lithium-heparin blood collection tube with an internal vacuum for suck the blood (BD Biosciences), serum of fetal bovine from Thermo Scientific Hyclone, Rosewell Park Memorial Institute-1640 (RPMI-1640) Medium from Mediatech, Inc. (USA), Phytohemagglutin (PHA) from Sigma-Aldrich (USA), antibiotic penicillin/streptomycin and separation medium for lymphocytes (LSM) obtained from Invitrogen (USA), 3H-thymidine obtained from Amersham (UK), filters made of glass fiber obtained from Conncetorate AG (Switzerland), trypan blue from Amresco (USA), phorbol-12-myristate-13-acetate (PMA) from MP Biomedicals (France), Streptavidin-HRP, clear high -binding, enzyme-linked immunosorbent assay (ELISA) kit and plate sealer, microplates made from polystyrene, reagent pack as a substrate containing H_2_O_2_ and TMB (Tetramethylbenzidine) from R&D system, Inc. (USA). Carbon tetrachloride (CCl_4_), and gelatin, olive oil from Sigma Chemical, Co. (USA), dimethyl sulfoxide (DMSO) from ThermoFisher Scientific (Loughborough, UK), for cell viability [3-(4,5-dimethylthiazole-2-yl)-2-5-diphenyltetrazolium bromide] (MTT) used obtained from ThermoFisher Scientific (Loughborough, UK).

### Lymphocyte Proliferation Assay Based on Radioactive ^3^H-Thymidine

For determination of lymphocyte proliferation assay, standard method of Froebel et al. (1999) was used. A written informed consent was obtained from the human volunteer for use of blood for experimental purposes. Blood was taken by puncturing the vein from a healthy volunteer’s human being for the separation of lymphocytes and poured into Lithium-heparin sterile tube having vacuum which then mixed properly. The study was conducted according to the guidelines of “World Medical Association” Declaration of Helsinki-ethical principles for medical research involving and was approved by the institutional Committee for Research Ethics “Centre of Biotechnology and Microbiology, University of Peshawar” approved vide number 9355/VC dated 12/12.2012. The blood mixed with a 2 mM L-glutamine poured in equal volume of 1640-RPMI in a tube of 50 ml sterile centrifuge. Diluted blood of 9 ml was poured on 5 mL LSM in 15 mL sterile centrifuge tube for layered. Care should be taken for not displace the two layered and centrifuged for 20 min at 25°C. Between the blood plasma and LSM phase mononuclear cells present in buffy layer was removed carefully into 15 mL sterile centrifuge tube containing insufficient RPMI-1640. At 4°C for 10 min the cells were washed at 300 × *g* by centrifugation. The peripheral blood mononuclear cells (PBMCs) in the form of pellets were re-suspended in RPMI-1640 containing 10% fetal bovine serum (FBS). The numbers of cells were estimated after trypan blue dilution at 1:1(v/v) on light microscope at 10X magnification. For proliferation assay, 3H-thymidine was diluted 1.0 μCi/ml to a 20 μCi/mL concentration with sterile RPMI-1640 and stored at -20°C in 5 ml aliquot. The dose effect of the test compounds in triplicate were mapped and labeled for assaying in sterile 96-well round bottomed plates. The PBMCs (1.2 × 10^5^ cells) at the concentration of 50 μL was grown with PHA of 50 μL to reach a concentration of 5 μg/ml, then added FBS RPMI-1640 and the test compounds of 50 μL made to a final concentration of 10 μg/mL. The culture was incubated for 72 h in a humidified atmosphere of 5% CO_2_ at 37°C. The 25 μL [methyl-3H] thymidine was added at 0.5 μCi in each well which was further kept for more 18 h. After incubation of mononuclear cells with radioactive 3H-thymidine was found to be incorporated into the DNA of dividing cells in each well determines the multiplication of T-cells. The cells harvester (Connectortae AG, Switzerland) was used for cell harvesting on glass filters. Vacuum suction was applied for drying the filters. The filter left for drying was then put into the scintillation tubes. Liquid scintillation called CytoScint was used to estimate the radioactivity as count per minute (cpm) by measuring the insertion of radioactive thymidine in the dividing cells and then put the tubes in a counter scintillation obtained from Beckman (USA).

### Determination of IL-2 Generation by Cell Culture

Fresh T-lymphocytes was used to investigate the effect of steroidal alkaloids on the production of IL-2. T-cells proliferation method was used for isolation of PBMCs from fresh venous blood. For this purpose 50 μL of cell suspension [2.5 × 10^6^ cell/mL, 50 μL of phytohemagglutinin (PHA) final concentration of 20 ng/mL], 50 μL of phorbolmyristate acetate (PMA, final concentration of 20 ng/mL), and 50 μL of the samples (final concentration of 0.5, 5.0, or 20 μg/mL were added in flat-bottomed 96-well plates. It was stored at 37°C for 18 h in a 5% CO_2_ incubator and ELISA was performed for IL-2 estimation from collected supernatants.

### Enzyme-Linked Immunosorbent Method for Interleukin 2

IL-2 ELISA Kit (ab174444) was used for determination of Interleukin-2. Recombinant anti-interleukin-2 was diluted to give a concentration of 4 μg/mL which was then used at 100 μL/well to stick in polystyrene flat-bottom micro-plates. The ELISA plate sealers were used for sealing the coated plates and stored at 25°C for 24 h. Buffer solution (0.05% Tween 20 in PBS, pH 7.2–7.4) of 300 μL were used three times to wash the plates and solution of antibody aspirated followed by the addition of 100 μL blocking buffer in each well and were kept at 25°C for 1 h.

### Estimation of IL-2 ELISA

The treated cells collected from supernatant were estimated for IL-2. The 100 μL of culture supernatant samples (1.0% bovine serum albumin, 0.05% polysorbate-20) in TBS were added in each 96-well micro-plates contained confined coated antibody as mentioned and were stored at 25°C for 2 h. To each well were added a 100 μL of 200 ng/mL goat biotinylated anti-human interleukin-2 antibody. Repeatedly washing was done followed by the addition of working solution of streptavidin-HRP of 100 μL in each well which was then incubated in the dark at room temperature for 20 min. Again the washing step was carried out and in each well a 100 μL of substrate solution was added and left at 25°C in dark for 20 min followed by the addition of 50 μL of stop solution in each well. The plate photometer was used at 450 nm to measure the optical density of each well.

### 3T3 Cells and MTT Cytotoxicity Assays

The 3T3 NIH mouse embryo fibroblast cells were used to perform *in vitro* cytotoxicity assays (Scudiero et al., 1988). The 96-well flat-bottomed plate containing 6 × 10^3^ cell/well in 100 μL complete media were used for MTT assay (ab112118) on 3T3 cells and were incubated in a 5% CO_2_ incubator at 37°C for 24 h. The media was replaced by media containing the test samples at different concentration (0.5, 5, and 50 μg/mL) and placed in a 5% CO_2_ incubator at 37°C for 48 h. The cell viability was checked for each test samples by using 0.5 mg/mL of MTT in complete media for 4 h. The supernatant was removed and added 100 μL of DMSO in each well to dissolve the formazan complex formed by the action of mitochondrial dehydrogenases and were observed at 540 nm for determination of optical activity. The result were examined and represented in mean ± SD by using SPSS software.

### Animal Study

A healthy male albino rats aged 2–3 months were fed standard rodent diet. There were six groups of rats and each group contains six animals which were kept in a light/dark cycle. The experimental study was approved by the animal ethical committee of the Center of Biotechnology and Microbiology, University of Peshawar. All animals received humane care and all protocols involving the animals were in compliance with the guidelines approved by the Institutional Ethics Committee of center of Microbiology and Biotechnology, University of Peshawar adhering to the guidelines of the Institutional Animal Care and Use Committee (IACUC) for animal studies.

### *In vivo* Study Design

Male albino rats of the normal control group received vehicle while CCl_4_ treated group were inject 0.5 ml/Kg CCl_4_ dissolve in olive oil intraperitoneally two times a day for 2 days. The positive control group was pretreated with silymarin at a dose of 200 mg/Kg (Ghayur and Gilani, 2006) and the other groups, were treated with sarcovagine-D, holaphylline, and alkaloid C, compounds at a dose of 20 mg/Kg, for 3 days prior of CCl_4_ injection (Thomas and Donald, 2005) and during the CCl_4_ injections for 2 days.

### Quantitative Evaluation of Liver Histopathology Assessment

After 24 h of last CCl_4_ injection, all experimental groups were dissected and the liver was cut into pieces were put in isotonic saline solution. The liver tissues were rapidly excised and fixed in neutral buffered formalin, dehydrated through a graded series of isopropyl alcohol, embedded in paraffin, and cut into 5 μm thick sections. The liver tissues were stained with hematoxylin-eosin (H&E). The liver tissues were then studied and examined under bright field microscope at different magnification using Nikon 90*i* microscope. Following under different condition histopathological analysis was carried out. The necrotic area was measured by in 20 different liver sections of each group using the NIS-elements software from Nikon, Japan. The damaged/injured area of the liver around the central vein was expressed in percentage compared to the whole area of the section.

### Immunohistochemistry

For immunohistochemistry, 4 μm thin liver sections were used. Briefly, the slides were deparaffinized in xylene and dehydrated in graded alcohol. The liver sections were incubated for 1 h with primary antibodies for liver macrophages, clone ED1 (clone ED1, abcam) (diluted 1:50). After thoroughly washing with PBS, the sections were then incubated with the secondary antibody, Texas Red-conjugated goat anti-mouse IgG (1:50) for 45 min. Then the slides were counterstained with DAPI, and mounted, while the expression profile and cellular localization of liver macrophages were analyzed by fluorescence microscopy (Nikon 90*i*, Japan).

### Biochemistry Study of Blood

To study the function and damage of liver, blood was collected from heart for serum and chemistry analyzer (Roche) were used to measure the level of ALT, AST, and ALP enzymes.

### MDA, SDA, and Glutathione Determination

To measure the liver antioxidant action, the animals were dissected and quickly excised the liver which was then frozen at -80°C for storage. The samples of hepatic tissue were melt and blended in equal volumes of cold phosphoric buffer saline at concentration of 50 mM (pH 7.4), 20 min centrifuged and kept at 4°C. Commercially available kit was used for MDA, SOD, GSH and protein from (Sigma-Aldrich, St. Louis, MO, USA).

### Statistical Analysis

Data analysis was carried out using SPSS software. Statistically significant differences between the samples were evaluated by one-way ANOVA. Values in the text are mean ± SD, standard deviation. Differences at *P* < 0.05, or *P* < 0.01 were considered as significant.

## Results

### Effect of Steroidal Alkaloid on T-Cell Proliferation

**FIGURE 1:**
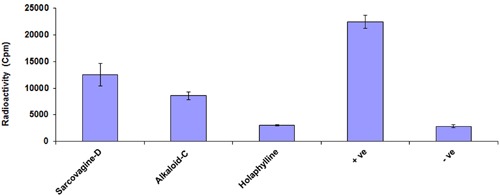
**Effect of steroidal alkaloid sarcovagine-D, alkaloid-C, and holaphylline on T-cells proliferation.** The purified compounds showed inhibitory activities of T-cell proliferation in the different range and compared with standard drug. Cells were incubated with 10 μg/mL concentrations of each of the compounds in RPMI media in the presence of 5 μg/mL PHA at 37°C for 72 h, and then pulsed with [3H]-thymidine. Cells were harvested and placed in 3 mL of β-scintillation liquid for counting. The percentage inhibition of each of the compounds was calculated using Microsoft Excel. Values are shown as mean ± SD of triplicate samples. The inhibited percentage of T-cell proliferation by 10 μg/mL standard drug cyclosporine was 99 ± 0.8. Positive control is the activated cells treated by PHA while negative control is the non-activated cells not treated with PHA.

The immunosuppressant drug (cyclosporine) administered for protection of organ transplant rejection by depressing the T-cells multiplication through reduced the production of IL-2. The T-cells proliferation inhibition also occurs by stopping the signal passage of IL-2 receptor through IL-2R antibodies. Therefore, first we investigate the effects of tested compounds on T-cells proliferation. We tested four mitogens to increase the efficiency of T-cells multiplication assay and PHA was among the good activator at 5 μg/mL (**Figure 1**). The inhibitions of pure samples were then checked on PHA and samples showed suppressive activity of T-cell with an IC50 value which is less than 10 μg/mL (**Figure 1**). Therefore, these compounds can be used as a drug for prevention of graft rejection. The steroid alkaloid sarcovagine-D, holaphylline, and alkaloid -C showed different result against T-cell proliferation. The pure compounds, sarcovagine-D (1) showed 78 ± 0.2, holaphylline (2) showed 95 ± 2.5, while alkaloid-C showed 82 ± 4.5 T-cell proliferation inhibitions, when used at less than 10 μg/mL concentration (**Table 1**).

**Table 1:** The effect of steroidal alkaloids from *Sarcococca saligna* on T-cells multiplication, IL-2 generation and cytotoxicity.

Tested compounds with standard drug	T-cell multiplication, % inhibition (mean ± SD) at 10 μg/ml	Inhibition of IL-2 generation, IC_50_ in μg/mL	3T3, IC_50_ in μg/mL
Sarcovagine-D	78 ± 4.5	2.95 ± 0.6	3.5 ± 0.3
Holaphylline	95 ± 1.2	1.35 ± 0.15	25 ± 1.7
Alkaloid-C	82 ± 0.8	0.9 ± 0.5	14 ± 1.8
Cyclosporine(Std 1)	99 ± 0.8	<0.05	-
Cyclohexamide (Std 2)	-	-	1.2 ± 0.4

The purified compounds showed inhibitory activities of T-cell proliferation in the range of 78 to 95% which are summarized in **Figure 1** and **Table 1**. Tacrolimus and cyclosporine used as a standard drug for comparison study.

### Effect of Steroidal Alkaloid on Generation of Interleukin-2

**FIGURE 2:**
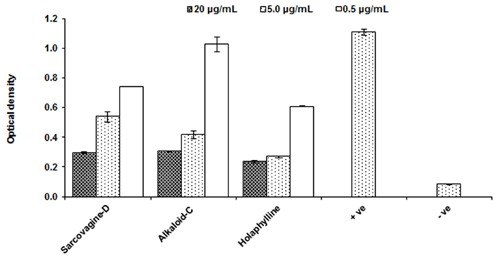
**Effect of purified test compounds on the generation of IL-2 production from T-lymphocytes at different concentration.** Human PBMCs were stimulated with PHA for 16 h in the presence or absence of the test compounds at 0.5, 5.0, and 20.0 μg/mL concentrations. IL-2 production was determined by ELISA and results are shown as mean ± SD for three observations. The IC50 of the standard cyclosporine is *P* < 0.05 μg/mL. PHA activated cells are positive control while PHA while non-activated and not treated cells with PHA is negative control.

The tested samples for inhibition of T-cells multiplications which is activated through the production of the cytokine IL-2 by PHA activated T-cells. The IL-2 is responsible for T-cells proliferation as well other immune cells which play role in cellular and adaptive immune response. All the tested compounds showed excellent suppressive effects on IL-2 production with an IC_50_ value less than 5.0 μg/mL as shown in **Figure 2** and **Table 1**.

#### Evaluation of Cytotoxicity

**FIGURE 3:**
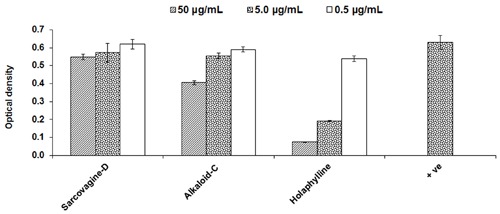
**Cytotoxic effects of steroidal alkaloids on 3T3 fibroblast cell line; positive control is not treated and consider as normal fibroblast.** Cells were incubated with compounds for 48 h then MTT was added for 4 h followed by addition of DMSO and absorbance was read at 540 nm using a 96-well plate reader. Results are expressed as mean ± SD of three replicates. Positive control is normal fibroblasts without any treatment.

Cytotoxicity of pure compounds was studied on mice fibroblast cell-lines (3T3) in order to examine the immunosuppressant action was not only due to their cellular toxicities. The compounds showed result and the IC_50_ value was found to be around 11.5 μg/mL except for sarcovagine-D. One compound holaphylline being considered as safe and have no cytotoxic effect on CTC cell lines up to concentration of 50 μg/mL (**Table 1** and **Figure 3**). Holaphylline was found to be less toxic and therefore was selected for *in vivo* testing. The chemical structure of sarcovagine-D, holaphylline, and alkaloid-C is described in **Figure 8**.

#### Effect of Biomarker Components of *S. saligna* on CCl_4_ as an Oxidative Inducer

**FIGURE 4:**
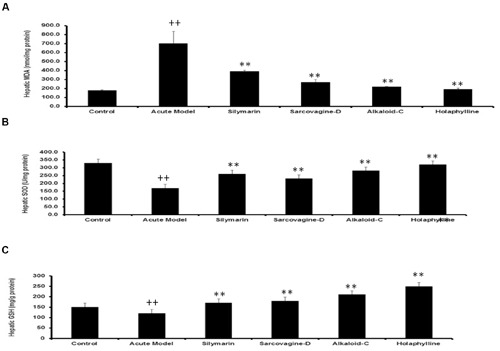
**(A)** Effects of *Sarcococca saligna* steroidal alkaloids (sarcovagine-D, alkaloid-C, holaphylline), on hepatic biochemical parameters: MDA **(A)**, GSH **(B)**, and SOD **(C)**, in CCl_4_-intoxicated rats. Data are expressed as the mean ± SD, *n* = 10. ^++^*P* < 0.01, when compared to the normal control; *P < 0.05, **P < 0.01, when compared to the CCl_4_ model control. Group I: normal control; Group II: CCl_4_ model control; Group III: 200 mg/kg silymarin+CCl_4_; Group IV: 20 mg/kg sarcovagine-D +CCl_4_; Group V: 20 mg/kg alkaloid-C +CCl_4_; GroupVI: 20 mg/kg holaphylline+CCl_4_.

To check the effect of pure compounds against CCl_4_-induced hepatic injury, malondialdehyde generation, glutathione level and superoxide dismutase enzymes level in the liver were estimated (**Figures 4A–C**). The level of MDA was drastically increased (*P* < 0.05) by CCl_4_ intoxication, however treatment with sarcovagine-D, holaphylline, and alkaloid-C has reduced the elevated level of MDA as shown (**Figure 4A**). In CCl_4_ intoxicated rats, the hepatic antioxidant enzyme SOD level dramatically decreased (*P* < 0.01), while the activities of antioxidant enzymes in the liver markedly decrease (*P* < 0.05) by co-treatment with pure compounds (**Figure 4B**). The GSH concentration in the rat liver were significantly decreased by intraperitoneal injection of CCl_4_ compared to control group (*P* < 0.01; **Figure 4C**). However, upon treatment with *S. saligna* biomarkers, the level of GSH was elevated (*P* < 0.05; **Figure 4C**).

#### Hepatoprotective Potential of *S. saligna* Pure Compounds: A Histological Studies

**FIGURE 5:**
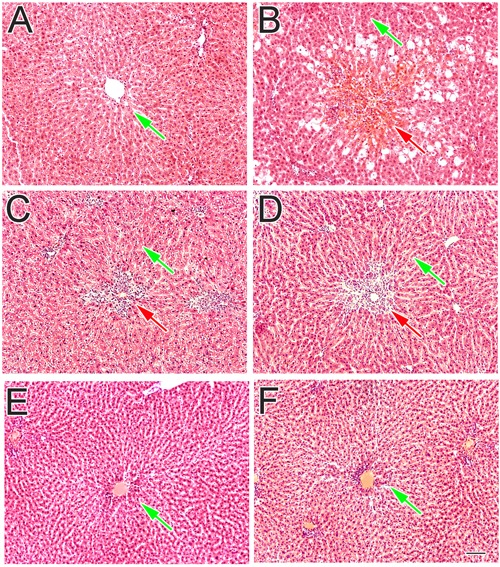
**The effect of test compounds on liver inflammation and its histopathological profile. (A)** Normal control group showed normal hepatocytes features: **(B)** CCl_4_ treated group showed the centrilobular necrosis: **(C)** Silymarin treated group: **(D)** sarcovagine-D: **(E)** alkaloid-C: and **(F)** holaphylline treated group against CCl_4_ oxidative hepatic injury. The red arrow showed the injured liver tissue while green arrows showed normal hepatic tissue.

The normal control rats liver which was dissect into sections were dye with reagent hematoxylin and eosin showed normal liver histology which is liver cords cells lined with endothelial cells with clearly defined curved area (**Figure 5A**). However on other side the CCl_4_-treated group liver sections showed declined production and damaged or injured hepatocytes containing hyaline bodies (**Figure 5B**). A lot of different inflamed cell penetrated, was available at the central vein space. In the space of damage place, liver injury was rare extended, especially soon at the side of lesion lined (**Figure 5B**). The shaped stability of the periportal and non-parenchymal cells were attained and decreased the pathological changes of CCl_4_ by treating standard drug silymarin at 200 mg/Kg as mentioned in **Figure 5C**. Instead of this some inflammatory cells were still found in the injured place around the central vein. However when the compound sarcovagine-D treated at 20 mg/Kg dose showed protection of liver membrane stability against CCl_4_ oxidative inducer injury and its appearance was normal as mentioned in **Figure 5D**. Similarly, other compounds holaphylline and alkaloid-C showed protection of liver in contrast to the CCl_4_ control group (**Figure 5F**). It also decreased the CCl_4_-induced pathological changes as the vicinity of sinusoidal lined with endothelial cells observed in normal liver (**Figure 5E**). Therefore, the compounds result showed dramatically decreased the diseases state induced by oxidative stress to the liver in contrast to the positive control group.

### Immunohistochemistry of Kupffer Cells of the Liver

**FIGURE 6:**
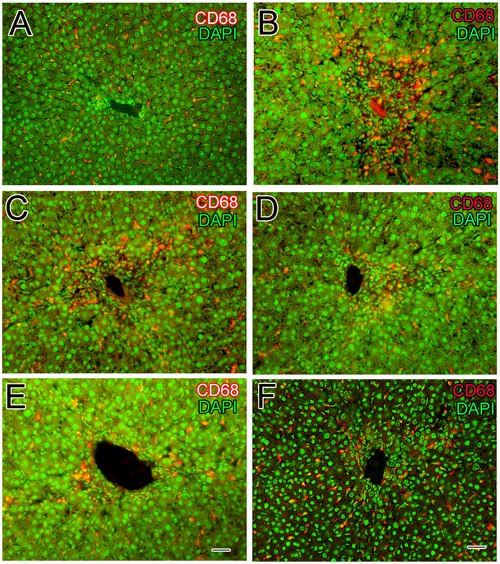
**Effects of steroidal alkaloids on hepatic macrophages (Kupffer cells).** Immunohistochemistry techniques showed CD68+Kupffer cells in the liver of normal control group around the central vein and the nucleus was stained with DAPI **(A)** and in the CCl_4_ induced liver injury showed numerous CD68+macrophages along the sinusoids around the central vein **(B)**. Silymarin treatment slightly reduced the activation of CD68+Kupffer cells to some level **(C)**. Sarcovagine-D and Alkaloid-C decreased the number of CD68+Kupffer cells to some extent, more or less similar to that of silymarin **(D,E)**. Holaphylline reduced the number of CD68+macrophages along the sinusoids around the central vein better than silymarin positive control **(F)**. Scale bar is 25 μm.

Inflammation of liver is associated with activation and migration of Kupffer cells into the hepatic cords of liver. Upon hepatic injury these macrophages secrete pro-inflammatory cytokines such as TNF-α and IL-6. In the normal control group, CD68+ immune-reactive cells with distinct slender nuclei were present in the sinusoidal spaces as well as a few around the central vein of liver (**Figure 6A**). The slender shaped nuclei of Kupffer cells were identified using DAPI staining as shown in **Figure 6A**. From fluorescence microscopy it is obvious that the resident macrophages having characteristic elongated shape in the sinusoidal spaces (**Figures 6A,B**). In CCl_4_ induced liver injury, the Kupffer cells were found to be densely stained and numerous in number (**Figure 6B**) in the injured area around the central vein. From DAPI staining, the nuclei of mixed inflammatory cells infiltrate was identified around the central vein (**Figure 6B**). Huge number of Kupffer cells was present around the injured central vein compared to normal control group (**Figure 6B**). Silymarin treatment has slightly reduced the number of activated Kupffer cells (**Figure 6C**) around the injured portion of liver compared to the CCl_4_ model group were further confirmed from the DAPI staining (**Figure 6C**). Interestingly, treatment with sarcovagine-D and alkaloid-C decreased (**Figure 6D**) the activated macrophages around the injured central vein (**Figure 6E**) to level similar like silymarin treatment but DAPI staining revealed inflammatory infiltrate around the central vein compared to normal control group (**Figures 6D,E**). However, treatment with holaphylline limited the activity of hepatic macrophages (**Figure 6F**), despite the CCl_4_ treatment as shown in the double channeled immunohistochemistry (**Figure 6F**). The macrophages were present in the sinusoidal spaces with distinct morphological features more in number compared to macrophages distribution in the normal control group as shown.

### Biochemical Studies of *S. saligna* Pure Compounds

**FIGURE 7:**
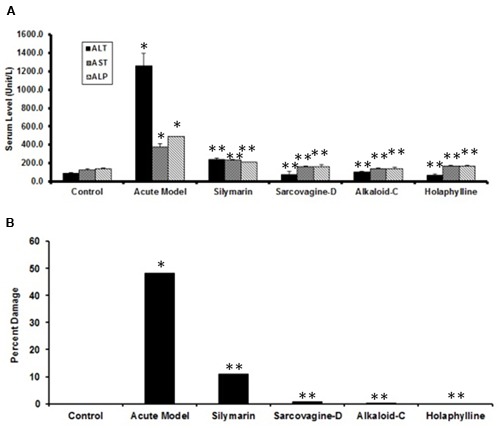
**(A)** Biochemical tests of the effects of *S. saligna* compounds on CCl_4_-induced liver injury: serum level of ALT, AST, and ALP as markers of liver injury under various conditions. Note that the sarcovagine-D, alkaloid-C, and holaphylline showed good protection (**P < 0.01) against CCl_4_-induced liver injury compared to CCl_4_ (*P < 0.05) or CCl_4_+silymarin (**P < 0.01). **(B)** Percent damage of the liver as assessed by histology under various conditions. Note the slight damage (12%) in the silymarin group whereas holaphylline showed minor damage up to (1%).

Biochemical studies were conducted to estimate the serum level of ALT, AST, and ALP in experimental groups. The group treated with CCl_4_ showed membrane injury and damage of hepatocytes (**Figure 7A**), also drastically increased the serum level of ALT, AST, and ALP. The level of ALT, AST, and ALP decreased by treated with standard silymarin but not to the optimum levels which showed that some hepatocytes necrosis still remained. However when treated with compounds sarcovagine-D, alkaloid,-C and holaphylline showed good hepatoprotective results and reduced the level of enzymes ALP, ALT, and AST better than silymarin standard drug as mentioned in **Figure 7A**. In the normal control group quantification of the histopathological data showed 0% pathology (**Figure 7B**) while CCl_4_ intraperitoneal injection caused huge pathological changes (48% damage) in the model group (**Figure 7B**). However, treatment with sarcovagine-D, alkaloid-C, and holaphylline at a dose of 20 mg per kg body weight restricted (*P* < 0.001) the pathological changes to 8, 4, and 1%, respectively. Therefore, holaphylline was better at hepatoprotection compared to silymarin which showed 12% damage (**Figure 7B**).

**FIGURE 8:**
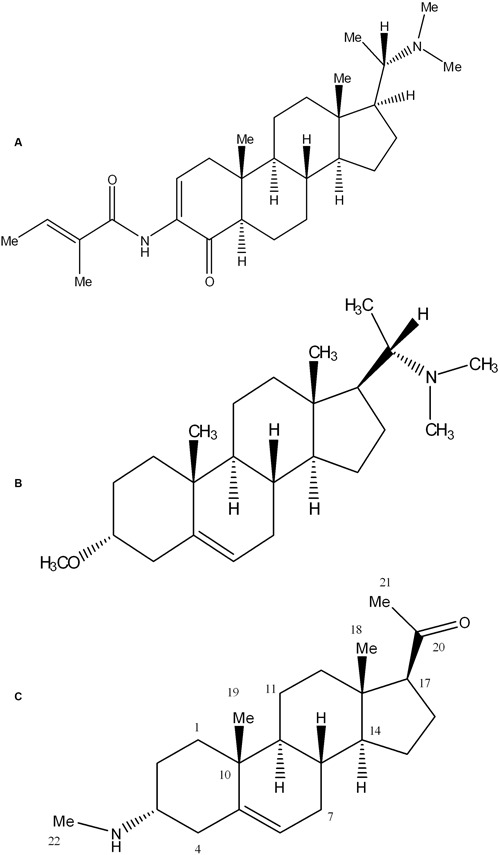
**The structures of the steroidal alkaloid compounds used in the present study: sarcovagine-D (A)**, alkaloid-C **(B)**, holaphylline **(C)**.

## Discussion

The immunity play has an important role to protect the body from any foreign particle or xenobiotics such as bacteria and virus which cause disease or potentially dangerous for body. During autoimmune diseases or organ transplanted from donor the body immune system recognize it as an outside tissue and start fight against its (Tilney and Kupiec-Weglinski, 1991). Leukocytes play an important role in the immunity process and kill any foreign tissue called body immune response (Akatsuka et al., 2003). White blood cells are different types and T-lymphocytes from it play an important role in all kind of immunity process (Chen et al., 2013).

The purpose of this biological assay was to investigate steroidal alkaloids that could be lead drugs to stop the proliferation of T-cells and having excellent property of hepatoprotective agents without causing cytotoxicty. As we know that damage of hepatocytes in viral hepatitis is because of immunity reaction and itself virus is not involved in it. We also check the impact of steroidal alkaloids on PHA-activated T-cells which activate specifically CD_4_ and T-cells (Wambre et al., 2012). The result of compounds showed inhibition against T-cells proliferation which was further study the effect of T-cells activated through PHA on generation of IL-2, which causes proliferation and as well other immunity cells multiplications. The CD_4_^+^ T-cells generated mostly IL-2 cytokines to different stimulating agent response through which activation of T-cells via receptor of T-cells and major histocompatibility complexes I and II antigen-presenting cells. The IL-2 cytokine is not detectable in normal healthy subject blood and its level rise drastically when a subject exhibit infection. The IL-2 generation increased rapidly by the PHA+PMA stimulation, reported in optimization protocol (Sullivan et al., 2000). The PHA and TCR responsible for crosslinking by binding sugar glycosidically on T-cells surface protein, which provokes signals 1 and 2 through linkage of co-stimulatory factor. All these activities take place on surface cell and consequently involve various signaling pathways. The phorbol 12-myristate 13-acetae (PMA) is structurally similar to a plant isolated compound Phorbol from *Croton tiglium*. PMA enter into cytoplasm l through cell membrane and activates protein kinase C enzyme as it structure is resemble to natural diacylglycerol PKC activator. When T-cells stimulated, generation of IL-2 start by PKC activation. The evaluation of steroidal alkaloid compounds is important for research as immunosuppressive compounds and showed excellent immunosuppressive properties (**Figure 6**).

We also evaluated compounds for cytotoxicity, which showed inhibition of T-cells proliferation and IL-2 production is not because of cytotoxicity. The steroidal alkaloids isolated from *S. saligna* have been used as a source of medicines for many diseases and serve as a basis for many pharmaceutical used (Newman and Cragg, 2007) and therefore these compounds were study for their anti-inflammatory effect.

The innate immune system activates after necrosis intensify the initial tissue injury during acute hepatitis and not necessarily cause the liver damage by inflammatory response. When the liver injured by CCl_4_ inducer, the aim of kupffer cell activation and hiring natural killer cells, neutrophils and monocytes in hepatic is to eliminate remove dead hepatocytes and this process is important for the reproduction of missed tissue, an examples are concanavalin-A (Nicoletti et al., 2000), lipopolysaccharide (Farghali et al., 2009) and acetaminophen hepatotoxicity, initiate the inflammatory response appearing neutrophils after liver injury in an hour and hiring the macrophages and monocytes within 24–48 h (Cover et al., 2006; Holt et al., 2008). The purified isolated steroidal alkaloids compounds from this plant showed positive protection results by depressing the injury to the hepatocytes without affecting more tissue in this *in vivo* study. The cytochrome P450 dependent monooxygenases activated metabolically by depositing CCl_4_ in the hepatocytes -the liver parenchymal cell to synthesize very high active metabolites, such as (CCl_3_OO^-^) and (CCl_3_^-^) radicals (Recknagel and Glende, 1973), which causing hepatotoxicity like liver cells death, degeneration and fibrosis (Weber et al., 2003; Manibusan et al., 2007). The generation of these free radical cause lipid oxidation by cover the cellular antioxidant defense system. In all this process the hepatic macrophages called kupffer cells play an important role in changing the severity of liver inflammation (Duffield et al., 2005; Friedman, 2005). It has been proposed that when liver injury occur, different pro inflammatory agents such as TNF-α and MCP are generated by kupffer cells, stimulated the stellate cells of liver to increase expression of extracellular matrix protein in chronic hepatic inflammation which utterly produce hepatic injury (Friedman, 2005; Gehring et al., 2006).

The finding of our study showed that the isolated steroidal alkaloid from *S. saligna* reduced liver inflammation by firstly reducing the T-cells multiplication and amount of IL-2 which change the entire inflammation reactions and as well non-cytotoxic, secondly acts as antioxidant and act as a free radicals scavenger which is produced by the hepatocytes. The *in vivo* study further showed that these steroidal alkaloids markedly decreased hepatic injury by CCl_4_-injury inducer and mixed inflammatory penetration. Therefore, we explored and suggest that steroidal alkaloids from *S. saligna* could be excellent immunosuppressive and hepatoprotective agents which have excellent therapeutic potential.

## Author Contributions

HA design and conducted the experiments and prepared the final version of manuscript. AmAl and AbAl helped in the analysis and interpretation of data as well as in preparing the initial draft of manuscript. AAd and NJ helped in the structure elucidation of compounds from *Sarcococca saligna*. BA and SA contributed in the collection of plant their extraction procedures and isolation of pure compounds while AJ assisted in manuscript writing and statistical analysis of the data. All authors read and approved the final manuscript.

## Conflict of Interest Statement

The authors declare that the research was conducted in the absence of any commercial or financial relationships that could be construed as a potential conflict of interest.
